# Devastating Postpartum Complications in an Adolescent Linked to New-Onset Inflammatory Bowel Disease and Antiphospholipid Antibody Syndrome

**DOI:** 10.1097/PG9.0000000000000105

**Published:** 2021-07-22

**Authors:** Anita Rao, Joseph Runde, Andrea D. Olivas, Tiffany Patton

**Affiliations:** From the * Section of Pediatric Gastroenterology, The University of Chicago Medical Center, Chicago, IL; † Section of Gastrointestinal and Hepatic Pathology, The University of Chicago Medical Center, Chicago, IL.

**Keywords:** inflammatory bowel disease, pregnancy, postpartum, colitis, venous thromboembolism, thrombosis, antiphospholipid, adolescent

## Abstract

Pregnancy can affect the severity of inflammatory bowel disease (IBD), and pregnant women with IBD are at a higher risk for venous thromboembolism compared with the general population. We report a previously healthy 16-year-old female who developed bloody diarrhea and venous thromboembolism following childbirth, with further evaluation revealing IBD and antiphospholipid antibody syndrome. This case highlights the impact pregnancy can have on IBD and other immunological disorders, and the potentially life-threatening risk of thrombosis in pregnant women with IBD.

## INTRODUCTION

Pregnancy is a unique immune-tolerant condition that can affect various immunological disorders including inflammatory bowel disease (IBD) (1). Also, as both pregnancy and IBD are risk factors for venous thromboembolism (VTE), pregnant women with IBD are known to have a higher VTE risk than the general population (2). This is the first reported case of IBD and antiphospholipid antibody syndrome (APLS) manifesting acutely after pregnancy. This case highlights the unpredictable effects pregnancy can have on the immune system and IBD, as well as the VTE morbidity risk for patients with IBD.

## CASE REPORT

A 16-year-old previously healthy gravida 1 para 1 female presented to the emergency department 8 days postpartum with sudden-onset right-sided weakness and inability to speak. Head computed tomography and angiography revealed left cerebral vein thrombosis resulting in a hemorrhagic stroke. She rapidly developed severe cerebral edema resulting in transtentorial herniation, and disseminated intravascular coagulation (DIC), evidenced by frank bleeding from the mouth, nose, and rectum, and laboratories including low fibrinogen <30 mg/dL and platelet count 55/nL with elevations of D-dimer >20 μg/mL and international normalized ratio 3.5. Hematochezia at this time was presumed to be secondary to DIC and improved with correction of the patient’s underlying coagulopathy, thus not triggering suspicion for colitis. She underwent an emergent hemicraniectomy with ventriculo-peritoneal shunt placement. On hospital day 5, she developed bilateral lower extremity deep vein thromboses. After extensive hematologic evaluation, a lupus anticoagulant/antiphospholipid syndrome (LA/APS) panel showed evidence for LA without anticardiolipin or anti-β2 glycoprotein antibodies, indicating APLS. LA/APS panels were repeated 4 times within 3 months confirming this result. Extensive rheumatologic evaluation including autoantibody and complement levels was negative, lowering the suspicion for systemic lupus erythematosus (SLE). She was initiated on warfarin anticoagulation and was discharged 2 months later to a chronic care facility.

**FIGURE 1.**
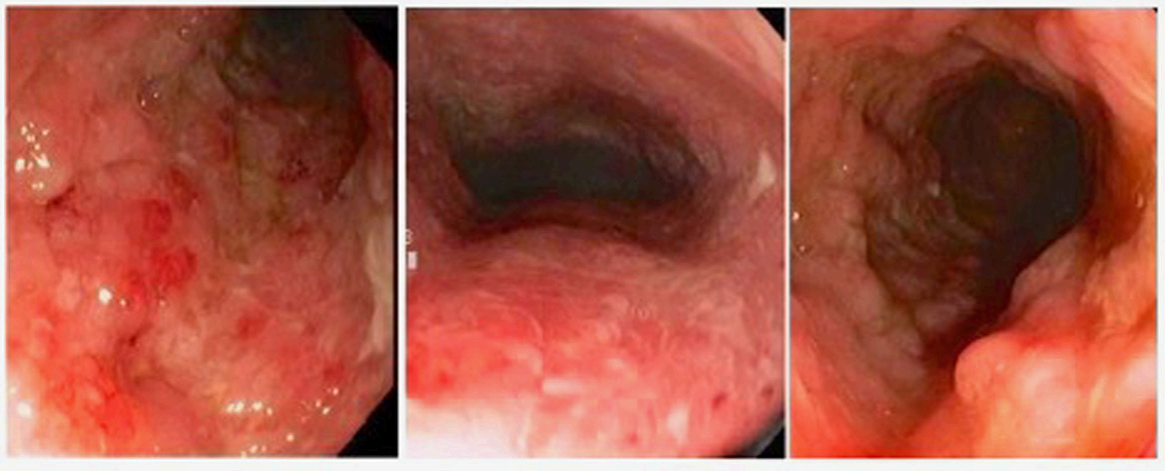
Colonoscopy with diffuse inflammation throughout the examined colon.

**FIGURE 2.**
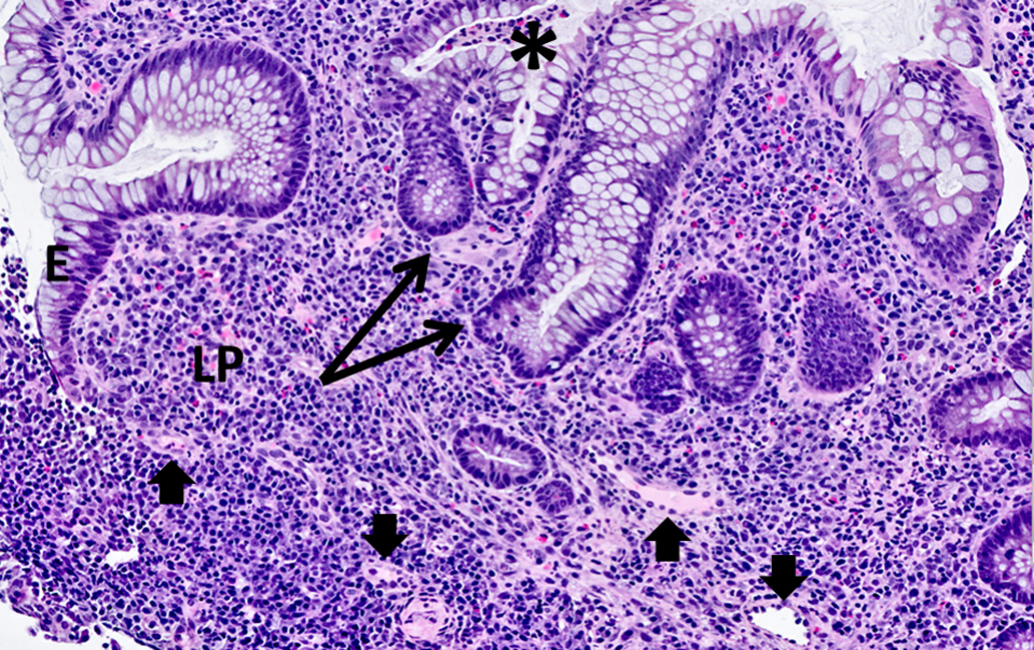
Colon biopsy. H&E-stained sections demonstrate LP expansion by a lymphoplasmacytic infiltrate, crypt architectural distortion (crypt branching, arrows), and neutrophils within the E with crypt abscess formation (*), consistent with active chronic colitis. Small blood vessels within the LP are free of thrombi (arrow heads). Histologic features of ischemic colitis, including mucosal atrophy and LP fibrosis, are absent. E = epithelium; H&E = hematoxylin & eosin; LP = lamina propria.

She represented to the pediatric intensive care unit 2 weeks later with hypovolemic shock, *Pseudomonas aeruginosa* urosepsis, and new-onset voluminous bloody diarrhea with an elevated international normalized ratio of 4.9. Anticoagulation was held. She was given several blood transfusions and underwent colonoscopy where histopathology showed diffuse moderate-to-severely active pancolitis with evidence of chronicity (Figs. 1 and 2). Video capsule endoscopy revealed a visually normal small bowel; the patient did not undergo small bowel enterography. Abdominal computed tomography angiography showed no evidence of mesenteric ischemia and was more indicative of an inflammatory process. Laboratory findings also suggested an inflammatory state, with elevated C-reactive protein 190 mg/L, erythrocyte sedimentation rate 66 mm/h, and fecal calprotectin >1000 μg/g. Complete blood count revealed mild leukocytosis with a white blood cell count 11.3/nL, anemia with hemoglobin 9.3 g/dL, and a normal platelet count. Albumin level was low at 2.0 g/dL. Stool tests for pathogens including *Clostridium difficile* were negative.

She was started on intravenous methylprednisolone and on significant improvement was transitioned to adalimumab and methotrexate for a new diagnosis of ulcerative colitis. Since the patient was staying at a long-term care facility, adalimumab was chosen for greater ease of administration over a medication that requires regular infusions. After 7 months of this regimen, fecal calprotectin was 244 μg/g and follow-up colonoscopy with histopathology revealed mildly active pancolitis, improved from prior. Currently, she has achieved clinical remission and continues prophylactic enoxaparin for APLS.

## DISCUSSION

We present a previously healthy young woman who developed thromboses and symptomatic colitis acutely following the birth of her child, with resulting evaluation indicating APLS and IBD. Interestingly, both APLS and IBD are chronic immune-mediated conditions that appeared to be precipitated by her pregnancy, as she previously had no symptoms of either. Pregnancy is an exceptional immune-tolerant condition in which nonself tissues are allowed to develop within the body without stimulating a detrimental immune response. An interesting feature of this condition is the effect of pregnancy on various immunological disorders, which may achieve remission or be exacerbated during pregnancy (1). IBD often affects women during their childbearing years, and 30%–40% of pregnant women with IBD will have worsening disease during pregnancy (1, 3). However, this case is unique in that our patient had no history of IBD or reported symptoms of IBD before her pregnancy. Additionally, although she developed hematochezia 8 days postpartum, IBD was not suspected at this time given that she was in DIC and her hematochezia resolved with correction of her coagulopathy. IBD was instead diagnosed during her second hospitalization.

Pregnancy is associated with VTE risk due to physiological changes including alterations in venous blood flow, mechanical obstruction by the gravid uterus, and vascular injury (4). Pregnant women with IBD are at an even higher risk for VTE stemming from the prothrombotic effects of IBD. According to a large population-based cohort study, pregnant women with IBD are 2-fold more likely to develop VTE than pregnant women without IBD (2). Thrombosis in IBD is an extraintestinal manifestation with high morbidity and mortality, and patients with IBD have a 3-fold higher risk of VTE than the general population (5). The pathogenesis of VTE in IBD is multifactorial and includes upregulation of inflammatory cytokines, acute phase reactants (including platelets), and procoagulants (i.e., factors V, VII, VIII, X, XI, XII, von Willebrand factor, and fibrinogen), and down-regulation of anticoagulants (including protein C and S), which all lead to a prothrombotic state (4).

Our patient additionally had APLS further contributing to thrombosis. APLS is an autoantibody-induced thrombophilia caused by antiphospholipid antibodies (APLA). APLS may be primary or secondary (in which it is associated with other autoimmune diseases such as systemic lupus erythematosus) (6). It is provoked by acute stressors such as pregnancy, and the manifestations of APLS in pregnant women include first trimester pregnancy loss, preeclampsia, and premature birth (7). Interestingly, IBD itself is associated with enhanced serological antibody formation including APLA (8). It is therefore possible that our patient had an immune predisposition to IBD with resulting serological APLA formation that was precipitated by her pregnancy. Her severe DIC and VTE raises suspicion for catastrophic antiphospholipid syndrome (CAPS), a life-threatening disease usually precipitated by an acute stressor (such as infection or pregnancy) resulting in multiorgan failure from extensive thrombosis in the presence of APLA (9, 10). While CAPS is still possible, the diagnosis requires histopathological evidence of small vessel thrombosis. This was not seen on our patient’s colonic biopsies (Fig. 2), which were instead far more consistent with IBD. Additionally, the gastrointestinal involvement seen in CAPS is usually ischemic colitis (10), but histopathology was inconsistent with this diagnosis (Fig. 2).

Our case highlights the impact that pregnancy can have on IBD and the potentially life-threatening risk of thrombosis in pregnant women with IBD. Additionally, the case emphasizes an association between IBD and APLS, which may lead to significant obstetric morbidity as well as VTE. It is prudent for practitioners to suspect IBD in patients with rectal bleeding and thrombosis and to consider APLS in patients with IBD who present with extensive thrombosis.

## ACKNOWLEDGMENTS

A.R., J.R., and T.P. equally contributed to the conception and design of the research. A.R. and A.D.O. contributed to the acquisition and analysis of the data. A.R., J.R., and T.P. contributed to the interpretation of the data. A.R. drafted the manuscript. A.R., J.R., A.O., and T.P. critically revised the manuscript. All authors agree to be fully accountable for ensuring the integrity and accuracy of the work; all have read and approved the final manuscript.
